# Virus-induced RGMa expression drives neurodegeneration in HTLV-1–associated myelopathy

**DOI:** 10.1172/jci.insight.184530

**Published:** 2025-04-24

**Authors:** Natsumi Araya, Makoto Yamagishi, Makoto Nakashima, Naomi Asahara, Kazuhiro Kiyohara, Satoko Aratani, Naoko Yagishita, Erika Horibe, Izumi Ishizaki, Toshiki Watanabe, Tomoo Sato, Kaoru Uchimaru, Yoshihisa Yamano

**Affiliations:** 1 Department of Rare Diseases Research, Institute of Medical Science, St. Marianna University School of Medicine, Kanagawa, Japan.; 2 Laboratory of Viral Oncology and Genomics, Department of Computational Biology and Medical Sciences, Graduate School of Frontier Sciences, The University of Tokyo, Tokyo, Japan.; 3 Neuroscience Unit Research Division, Mitsubishi Tanabe Pharma Corporation, Kanagawa, Japan.; 4 LSI Medience Co., Tokyo, Japan.; 5 Laboratory of Tumor Cell Biology, Department of Computational Biology and Medical Sciences, Graduate School of Frontier Sciences, The University of Tokyo, Tokyo, Japan.; 6 Department of Hematology/Oncology, and; 7 Department of Neurology, St. Marianna University School of Medicine, Kanagawa, Japan.

**Keywords:** Infectious disease, Neuroscience, Neurodegeneration, T cells, Transcription

## Abstract

Human T-lymphotropic virus type 1–associated (HTLV-1–associated) myelopathy (HAM, also known as tropical spastic paraparesis) is a rare neurodegenerative disease with largely elusive molecular mechanisms, impeding targeted therapeutic advancements. This study aimed to identify the critical molecule responsible for neuronal damage in HAM, its source, and the regulatory mechanisms controlling its expression. Utilizing patient-derived cells and established cell lines, we discovered that HTLV-1 Tax, in conjunction with specificity protein 1 (Sp1), enhanced the expression of repulsive guidance molecule A (RGMa), a protein known to contribute to neuronal damage. RGMa expression was specifically upregulated in HTLV-1–infected cells from patients with HAM, particularly in those expressing HTLV-1 Tax. Furthermore, in CD4^+^ cells from patients with HAM, the level of H3K27me3 methylation upstream of the *RGMA* gene locus was reduced, making *RGMA* more prone to constitutive expression. We demonstrated that HTLV-1–infected cells in HAM inflict neuronal damage via RGMa. Crucially, the neutralizing antibody against RGMa, unasnemab (MT-3921), effectively mitigated this damage in a dose-responsive manner, highlighting RGMa’s pivotal role in neuronal damage and its potential as a therapeutic target for alleviating neuronal damage in HAM.

## Abstract 2

HTLV-1-induced RGMa expression causes neuronal damage in HAM; neutralizing RGMa with unasnemab mitigates damage, offering a new therapeutic target for this neurodegenerative disease.

## Introduction

Recent advancements in molecular analysis techniques have renewed interest in infectious agents as potential causes of immune-mediated neurodegenerative disorders such as Alzheimer disease, Parkinson disease, amyotrophic lateral sclerosis, and multiple sclerosis (1, 2). However, the molecular mechanisms underlying nerve tissue damage caused by infectious agents remain largely unknown, hindering targeted therapy development. Human T-lymphotropic virus type 1–associated (HTLV-1–associated) myelopathy (HAM, also known as tropical spastic paraparesis, TSP) is a notable example of a neurodegenerative disorder hypothesized to result from an immune response triggered by infectious agents rather than direct neuronal infection. Affecting an estimated 0.25% to 3% of over 10 million HTLV-1–infected individuals globally, HAM leads to progressive and irreversible impairment, presenting a substantial unmet medical need.

Pathologically, spinal cord lesions in patients with HAM exhibit inflammatory cell infiltration, predominantly involving CD4^+^ T cells harboring HTLV-1–infected cells, HTLV-1 Tax–specific CD8^+^ T cells, and macrophages around blood vessels (3, 4). These cells are sources of inflammatory cytokines such as IL-1β, IFN-γ, and TNF-α (5). Moreover, IFN-γ stimulates astrocytes to produce excess CXCL10 chemokine, facilitating the migration of inflammatory cells into spinal cord lesions and perpetuating inflammation (6). Importantly, CXCL10 levels in cerebrospinal fluid correlate with the levels of neurofilament light chain (NF-L), a marker of neuronal damage, and are strongly associated with disease progression (7, 8). Consequently, the primary pathogenic process in HAM is believed to involve neural tissue damage resulting from the immune response initiated by the infiltration of HTLV-1–infected cells. Although targeting these infected cells has been proposed as a therapeutic strategy, which has resulted in symptom improvement in some instances, its effectiveness is limited in the context of advanced neurological damage (9, 10). This underscores the importance of understanding the molecular mechanisms underlying nerve damage and developing improved or alternative therapeutic approaches to overcome HAM.

Proposed mechanisms for neurological damage in HAM encompass 3 hypotheses: direct infection of central nervous system–resident (CNS-resident) cells by HTLV-1, molecular mimicry of host antigens by HTLV-1, and bystander damage (11). There is no convincing evidence to support the infection of CNS-resident cells or antigen mimicry, leaving bystander damage as the predominant hypothesis (11). In this bystander hypothesis, neurons and glial cells suffer damage from toxic or inflammatory products released by HTLV-1–infected T cells and the subsequent immune response within the CNS. Nevertheless, this bystander damage hypothesis remains speculative (11).

This study aimed to elucidate the mechanism underlying neuronal damage in HAM by coculturing neurons with PBMCs derived from patients with HAM and evaluating the extent of neuronal damage based on the release of NF-L from neurons as an indicator. The results demonstrate that cellular components from patients with HAM exhibited neuronal damage effects. Further analysis revealed that HTLV-1 Tax induces the expression of repulsive guidance molecule A (RGMa), a protein associated with neuronal damage, and its expression was specifically upregulated in HTLV-1–infected cells from patients with HAM. This implicates HTLV-1–infected cells from patients with HAM in directly inducing neuronal damage via RGMa. Additionally, inhibiting the action of RGMa shows promise as a potential therapeutic strategy for mitigating neurological damage in HAM.

## Results

### Neuronal cell damage induced by HAM-PBMCs.

**Figure 1:**
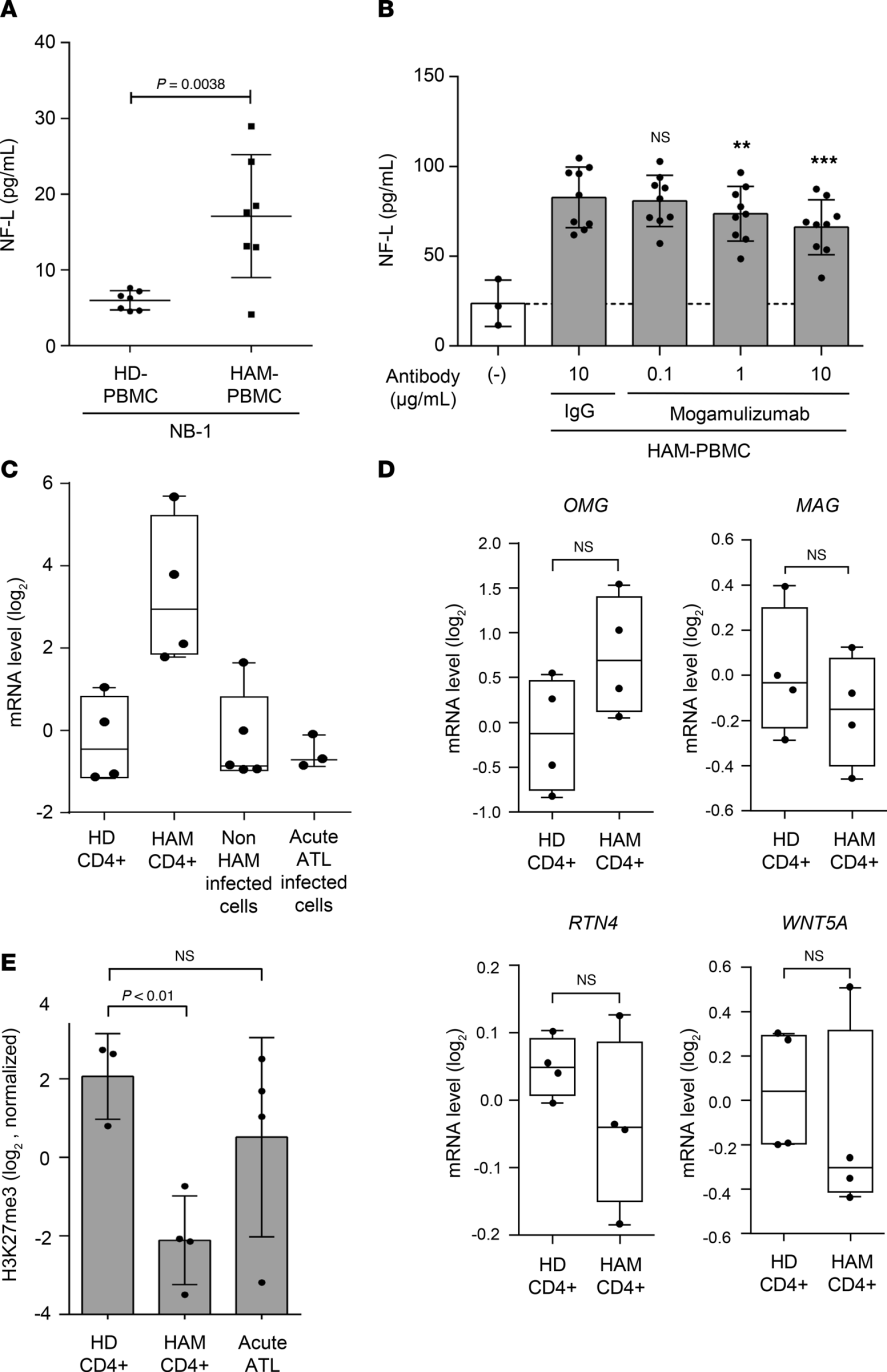
Neuropathic effects of HAM-PBMCs. (**A**) The concentration of NF-L (pg/mL) in the supernatant when NB-1 cells were cocultured with HAM-PBMCs (*n* = 7) or HD-PBMCs (*n* = 7). (**B**) The concentration of NF-L (pg/mL) in the supernatant when NB-1 cells were cocultured with HAM-PBMCs (*n* = 9) and with mogamulizumab (antiCCR4) in a dose-dependent manner for 72 hours. (**C**) The comparison of *RGMA* mRNA gene expression levels using DNA microarray among normal CD4^+^ T cells (HD CD4^+^: *n* = 4), HAM patient–derived CD4^+^ T cells (HAM CD4^+^: *n* = 4), ACs (*n* = 2), and smoldering/chronic-type-ATL patient–derived (*n* = 3) HTLV-1–infected CD4^+^ T cells (Non-HAM infected CD4^+^ T cells: *n* = 5), and acute-type-ATL patient–derived HTLV-1–infected CD4^+^ T cells (Acute ATL infected cells: *n* = 3). (**D**) The comparison of the expression levels of the genes associated with the inhibition of neuroregeneration (*OMG*, *MAG*, *RTN4*, and *WNT5A*) between HD CD4^+^ (*n* = 4) and HAM CD4^+^ T cells (*n* = 4). (**E**) The enrichment levels of H3K27me3 –2916 bp upstream from the TSS of the *RGMA* gene locus in HD CD4^+^ (*n* = 3), HAM CD4^+^ (*n* = 4), and acute-ATL infected cells (*n* = 4). Data are shown as mean ± SD. **P < 0.01; ***P < 0.001 by unpaired *t* test (**A** and **D**) or 1-way ANOVA with Dunnett’s multiple-comparison test (**B**, **C**, and **E**). NF-L, neurofilament light chain.

To investigate the potential neurotoxic effects of PBMCs derived from patients with HAM (HAM-PBMCs), we measured the concentration of NF-L in the supernatant when the human neuronal cell line NB-1 was cocultured with HAM-PBMCs (*n* = 7) in comparison to coculture with PBMCs from healthy donors (HD-PBMCs, *n* = 7). The concentration of NF-L in the culture supernatant was measured as a marker for neuronal damage. Results showed a significantly increased concentration of NF-L in cocultures with HAM-PBMCs compared with those with HD-PBMCs (*P* = 0.0038, Figure 1A), indicating a neurotoxic effect exerted by HAM-PBMCs.

To ascertain the importance of HTLV-1–infected T cells among HAM-PBMCs in causing neuronal cell damage, we measured the NF-L concentration in the medium of NB-1 cells cocultured with HAM-PBMCs in the presence or absence of an anti-CCR4 antibody (mogamulizumab), which is known to deplete HTLV-1–infected T cells (12, 13). In vitro treatment with mogamulizumab significantly reduced the NF-L concentration compared with the negative control (human IgG), indicating the critical role of HTLV-1–infected T cells in HAM-PBMCs in inducing neuronal cell damage (Figure 1B).

To identify the causative factors behind the observed neuronal cell damage induced by HAM-PBMCs, we conducted a comprehensive analysis of gene expression profiles in HAM CD4^+^ T cells, the major reservoir of HTLV-1, using DNA microarray. Among the genes associated with the inhibition of neuroregeneration via Rho signal transduction pathways (14, 15), the expression levels of *RGMA* were significantly elevated solely in HAM CD4^+^ T cells when compared with CD4^+^ T cells from HDs (non–HTLV-1–infected CD4^+^ T cells), HTLV-1–infected cells from asymptomatic carriers (ACs), and patients with smoldering/chronic-type adult T cell leukemia (ATL), as well as HTLV-1–infected cells from patients with acute ATL (*P* < 0.05) (Figure 1C). Additionally, in previously reported transcriptome data (16), *RGMA* gene expression was found to be elevated in HAM CD4^+^ T cells compared with CD4^+^ T cells from HDs (data not shown). However, the expression levels of other related genes such as oligodendrocyte myelin glycoprotein (*OMG*), myelin-associated glycoprotein (*MAG*), reticulon 4 (*RTN4*), and *WNT5A*, did not exhibit significant differences between HAM CD4^+^ T cells and HD CD4^+^ T cells (Figure 1D). In our pursuit of understanding the mechanism behind the specific expression of *RGMA* in HAM CD4^+^ T cells, we investigated histone H3 lysine 27 trimethylation (H3K27me3), a known gene-silencing marker, at the promoters of all genes via H3K27me3 ChIP-on-chip. The analysis of H3K27me3 levels at the *RGMA* promoter locus, situated –2916 bp upstream from the transcription start site (TSS), revealed a significant decrease in H3K27me3 levels in HAM CD4^+^ T cells compared with HD CD4^+^ T cells and acute-type ATL cells (Figure 1E). This finding suggests that the *RGMA* gene is expressed in HTLV-1–infected CD4^+^ T cells from patients with HAM through epigenetic deregulation of H3K27me3.

### Elevated RGMa expression in HTLV-1–infected T cells of patients with HAM.

**Figure 2:**
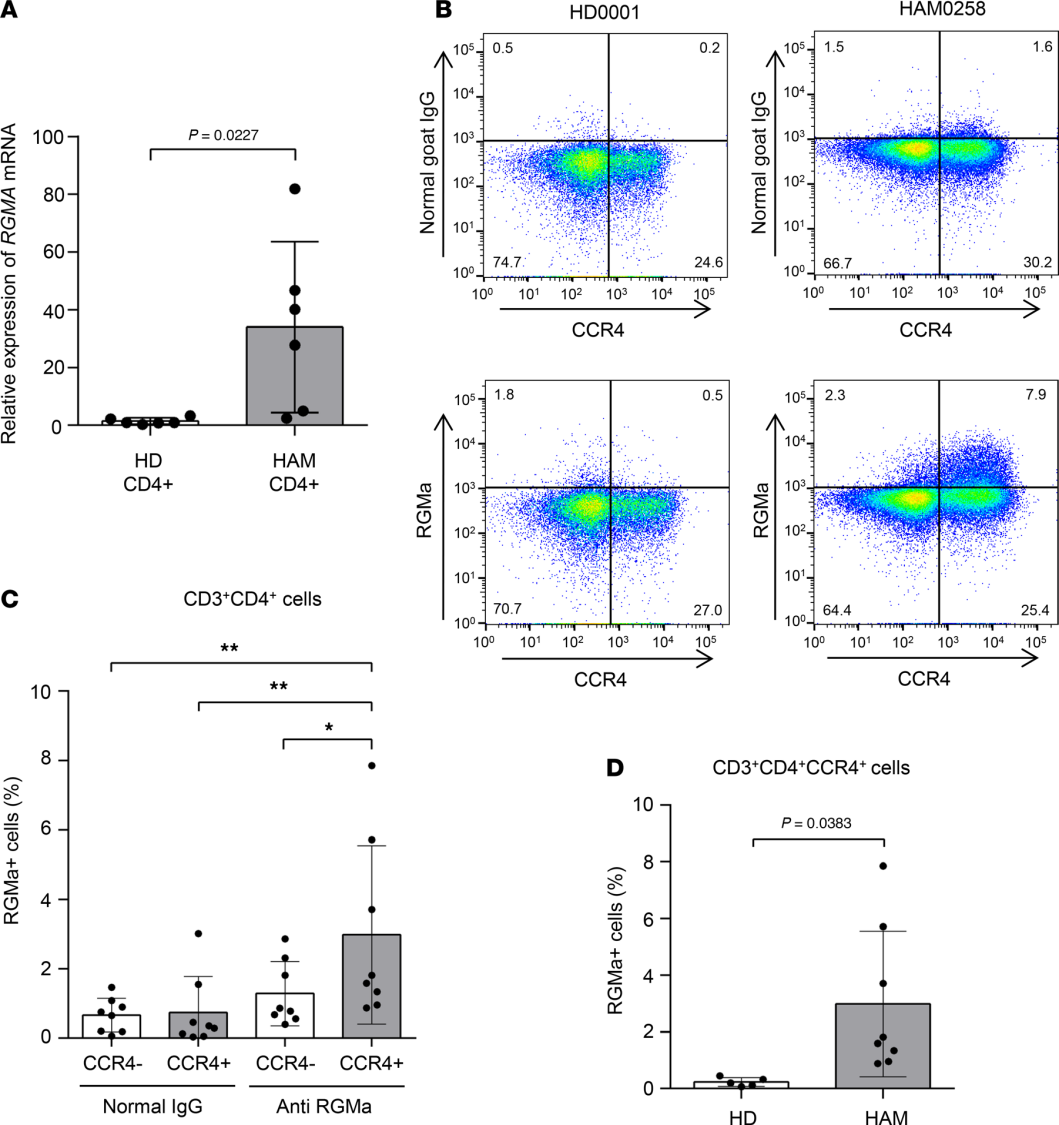
An increase in *RGMA* gene expression levels in HAM CD4^+^ T cells. (**A**) The validation of *RGMA* mRNA gene expression levels using qRT-PCR in HD CD4^+^ (*n* = 6) and HAM CD4^+^ T cells (*n* = 6). (**B**) Expression of RGMa protein in CD3^+^CD4^+^CCR4^+^ T cells from HAM-PBMCs. Representative dot plots of CCR4 and normal goat IgG (upper) or RGMa expression (bottom) in CD3^+^CD4^+^ gated cells from HD-PBMCs (left) or HAM-PBMCs (right) cultured for 2 days. (**C**) Graph shows the percentage of RGMa protein–expressing cells in CCR4^–^ cells or CCR4^+^ cells in CD3^+^CD4^+^ gated cells from HAM-PBMCs (*n* = 8) cultured for 2 days, compared with the isotype control, normal goat IgG. (**D**) Graph shows the percentage of RGMa protein–expressing cells among CD3^+^CD4^+^CCR4^+^ gated cells from HD-PBMCs (*n* = 5) or HAM-PBMCs (*n* = 8) cultured for 2 days. Data are shown as mean ± SD. *P < 0.05; **P < 0.01 by unpaired *t* test (**A** and **D**) or 1-way ANOVA with Dunnett’s multiple-comparison test.

To further validate the elevated expression levels of *RGMA* in HAM CD4^+^ T cells, we conducted quantitative reverse transcriptase PCR (qRT-PCR). The results confirmed a significant 16.4-fold increase in *RGMA* mRNA expression in HAM CD4^+^ T cells (*n* = 6) compared with HD CD4^+^ T cells (*n* = 6) (*P* = 0.0227, Figure 2A). Subsequent efforts to verify RGMa protein expression through flow cytometric analysis of ex vivo PBMCs derived from patients with HAM did not demonstrate an increase in expression (data not shown). This led to the hypothesis that RGMa protein expression may become more apparent following the in vitro culture of HAM-PBMCs. Flow cytometric analysis of PBMCs from HDs or patients with HAM cultured for 2 days revealed observable RGMa protein expression in HAM CD4^+^ cells, notably within cells characterized by the HTLV-1 infection marker CCR4 (Figure 2B). This finding was supported by multiple HAM-PBMC samples (Figure 2C), showing that RGMa expression was predominantly elevated in CD4^+^CCR4^+^ cells as compared with CD4^+^CCR4^–^ cells, with a significant variance in the percentage of RGMa-expressing cells (CCR4^–^ cells = 1.288% ± 0.923%, CCR4^+^ cells = 2.984% ± 2.569%, *P* < 0.05; Figure 2C). Moreover, the percentage of RGMa protein–expressing CD4^+^CCR4^+^ cells from patients with HAM was significantly higher than that observed in cells from HDs (HD = 0.231% ± 0.156%, HAM = 2.984% ± 2.569%, *P* = 0.0383; Figure 2D). These findings indicate a marked increase in RGMa expression levels specifically in HTLV-1–infected T cells from patients with HAM, with further elevation upon cell culture.

### HTLV-1 Tax–dependent induction of RGMa.

**Figure 3:**
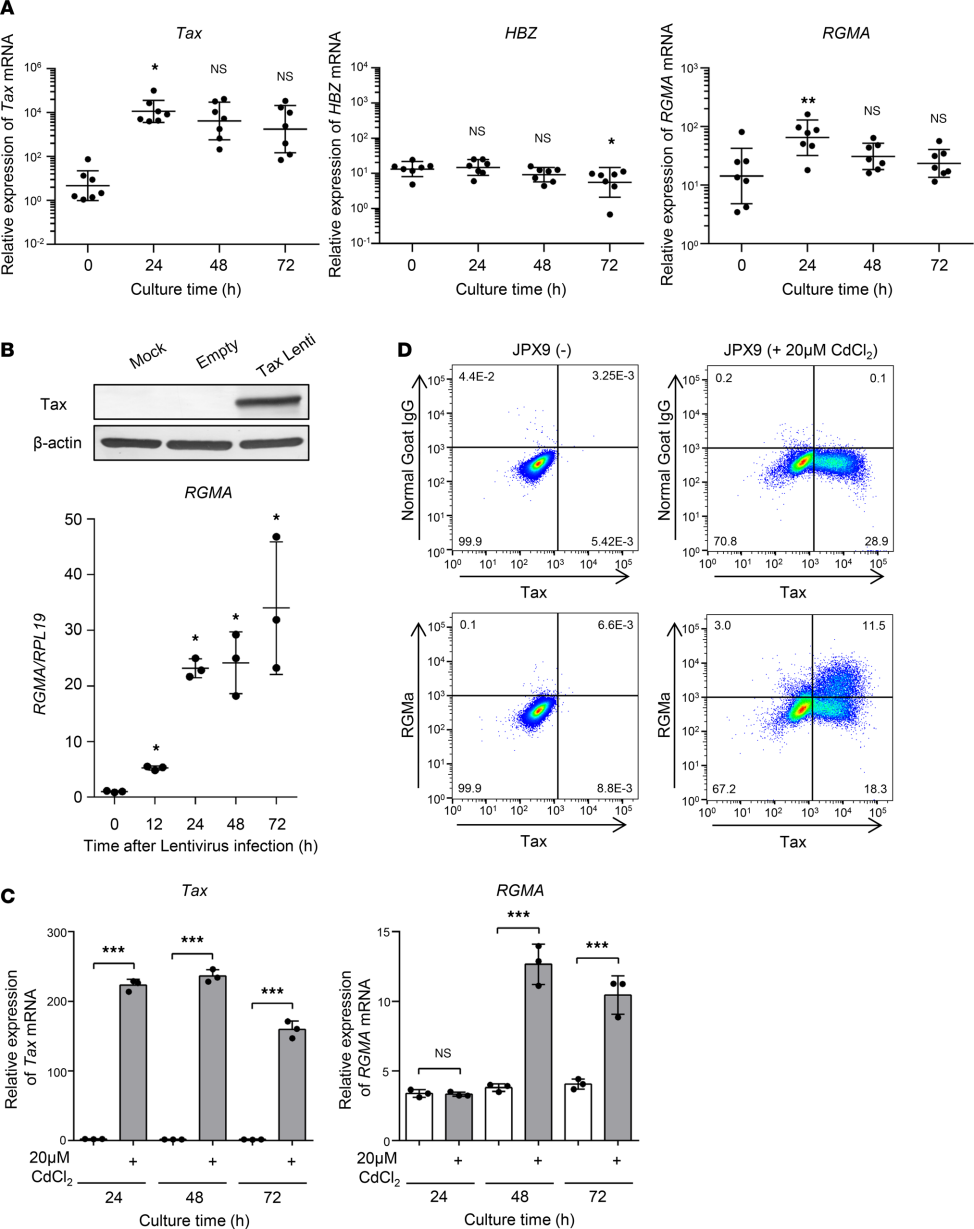
*RGMA* gene induction is HTLV-1 Tax dependent. (**A**) *Tax* (left), *HBZ* (middle), and *RGMA* (right) gene expression levels in cultured HAM-PBMCs (*n* = 7) in a time-dependent manner. *RPL19* was used as an internal control. (**B**) Tax-dependent *RGMA* mRNA gene induction in Jurkat cells, which were infected with lentivirus carrying the *Tax* gene. Top: Tax expression in the Jurkat cells was confirmed by Western blotting. β-Actin was measured as an internal control. Bottom: The induction levels of the *RGMA* gene were evaluated by qRT-PCR in a time-dependent manner (*n* = 3). (**C**) *Tax*-dependent *RGMA* mRNA gene induction in JPX9 cells treated with 20 μM CdCl_2_ in a time-dependent manner. *Tax* mRNA (upper) and *RGMA* mRNA (bottom) were measured by qRT-PCR (*n* = 3). *GAPDH* was measured as an internal control. (**D**) Tax-dependent RGMa protein induction in JPX9 cells treated with 20 μM CdCl_2_ for 3 days. Dot plots of Tax and normal goat IgG (upper) or RGMa expression (bottom) in JPX9 cells. JPX9(-), untreated JPX9 cells; 20 μM CdCl_2_ JPX9, CdCl_2_-supplemented JPX9 cells. Data are shown as mean ± SD. *P < 0.05; **P < 0.01; ***P < 0.001 by 1-way ANOVA with Dunnett’s multiple-comparison test (**A**), 2-sided Student’s *t* test (**B**), or an unpaired *t* test (**C**). Experiments were performed in triplicate (**B** and **C**).

Given that in vitro culture of HAM-PBMCs is known to induce the expression of HTLV-1–encoded genes, particularly HTLV-1 *Tax* (13), we examined the relationship between the expression levels of the *RGMA* gene and those of HTLV-1−encoded genes, *Tax* and *HBZ*, under cell culture conditions using qRT-PCR. A significant elevation in *RGMA* gene expression was observed after 24 hours of incubation, alongside an increase in *Tax* gene expression. Conversely, *HBZ* gene expression did not exhibit a significant increase after 24 hours (Figure 3A). *RGMA* and *Tax* gene expression demonstrated a decrease after 48 and 72 hours relative to their levels at 24 hours, suggesting a link between *Tax* and *RGMA* gene induction.

To elucidate whether Tax could directly induce *RGMA* gene expression, we constructed a Tax-inducing lentiviral vector and integrated *Tax*-coding cDNA into Jurkat cells, a T cell acute lymphoblastic leukemia cell line. Western blotting confirmed the induction of Tax protein in Jurkat cells (Figure 3B). Introduction of Tax via lentivirus led to a time-dependent increase in *RGMA* gene expression in Jurkat cells (*P* < 0.05) (Figure 3B). This finding was further explored using the JPX9 cell line, which expresses Tax protein upon cadmium chloride (CdCl_2_) treatment. Treatment of JPX9 cells with CdCl_2_ resulted in a significant increase in *Tax* gene expression after 24 hours, followed by an elevation in *RGMA* gene expression after 48 hours (Figure 3C). Additionally, flow cytometric analysis of JPX9 cells 3 days after CdCl_2_ addition revealed specific RGMa protein expression in the Tax-expressing cell population (Figure 3D).

### Tax and Sp1 cooperatively activate RGMA transcription via upstream regulatory elements.

**Figure 4:**
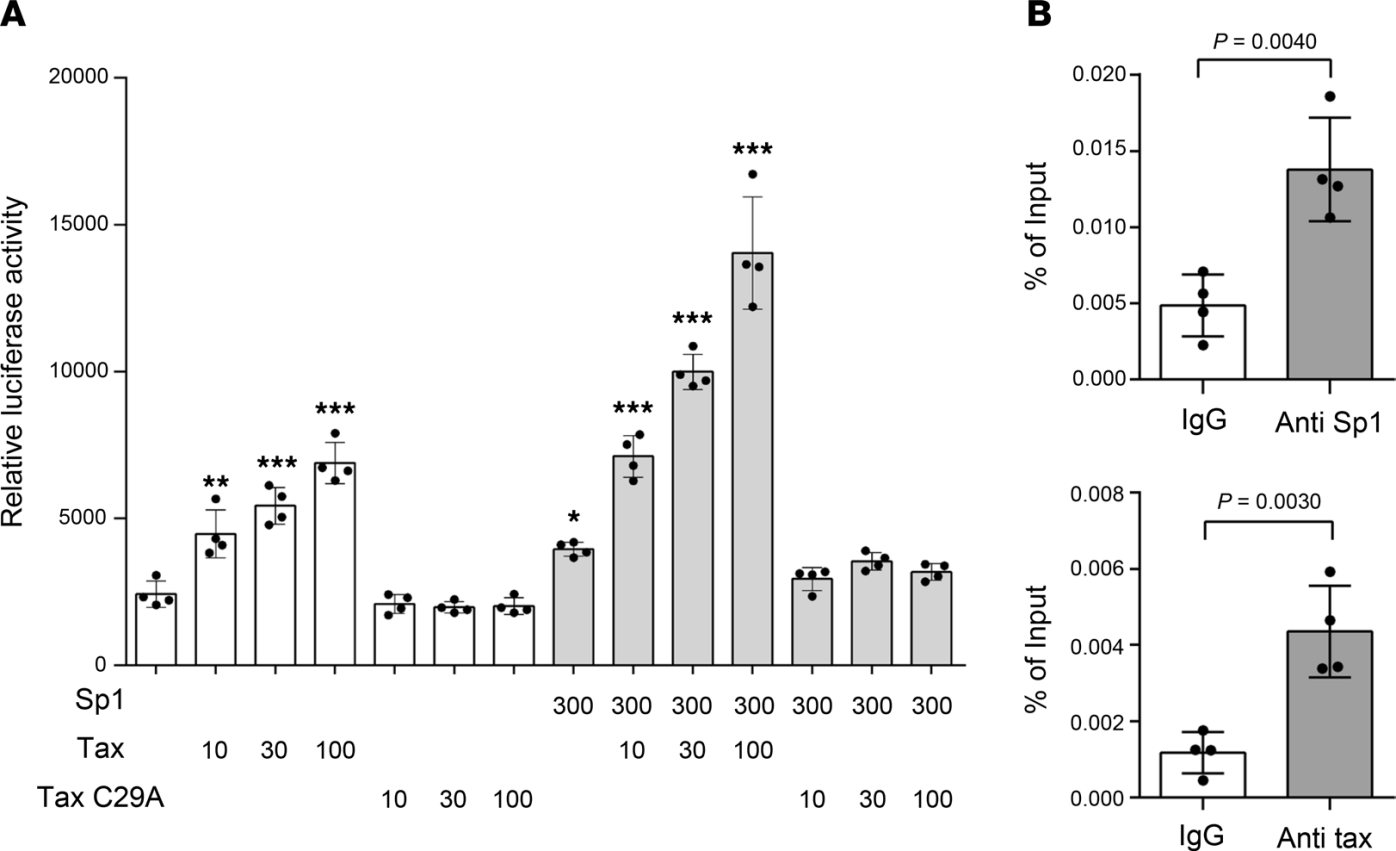
Tax and Sp1 cooperatively enhance *RGMA* transcription. (**A**) Coactivation of the *RGMA* transcriptional regulatory region by Sp1 and Tax. HEK293 cells were transfected with 10 ng of *RGMA*-Luc reporter plasmid, 300 ng of Sp1 expression plasmid, and 0–300 ng of either Tax or Tax C29A expression plasmid, as indicated. Values were normalized to β-galactosidase activity as an internal control (*n* = 4). (**B**) ChIP-qPCR analysis of the predicted Sp1 binding region upstream of the *RGMA* locus in JPX9 cells following treatment with 20 μM CdCl_2_ for 48 hours. Tax and Sp1 binding were evaluated in comparison to mouse IgG and rabbit IgG, respectively. The immunoprecipitated DNA was normalized to input DNA (*n* = 4). Data are shown as mean ± SD. *P < 0.05; **P < 0.01; ***P < 0.001 by 1-way ANOVA with Dunnett’s multiple-comparison test (**A**) or 2-sided Student’s *t* test (**B**). Experiments were performed in triplicate.

We hypothesized that a region approximately 2916 bp upstream of the *RGMA* gene, where H3K27me3 modification is characteristically removed in HAM-CD4^+^ T cells (Figure 1E), acts as a regulatory domain for *RGMA* gene expression. Given our previous report that Tax forms a complex with specificity protein 1 (Sp1) to synergistically activate the transcription of target genes (13), we employed the transcription factor binding prediction software JASPAR (17) to analyze Sp1 binding sites within the region from –3016 bp to –2816 bp. This analysis identified 4 Sp1 binding sites (–3007 to –2997 bp, –3006 to –2998 bp, –2986 to –2976 bp, and –2999 bp to –2991 bp) (Supplemental Figure 1; supplemental material available online with this article; https://doi.org/10.1172/jci.insight.184530DS1). Subsequently, we performed reporter assays using cells transfected with the *RGMA*-Luc luciferase reporter plasmid, which encompasses the –3500 to +100 bp region of the *RGMA* gene, to investigate the effects of Sp1 and Tax on the transcriptional activation of *RGMA*. The data showed that cotransfection with Sp1 activated the luciferase reporter, and further cotransfection with Tax synergistically increased the reporter activity in a Tax concentration–dependent manner. Importantly, the reporter activation by Tax was not observed with the nuclear localization–deficient Tax C29A mutant (18), demonstrating that Tax activates the *RGMA* transcriptional regulatory region within the nucleus (Figure 4A). Moreover, we evaluated whether Tax and Sp1 localized to the region predicted by JASPAR using ChIP-qPCR with JPX9 cells expressing Tax (amplification region: –3028 bp to –2879 bp from the *RGMA* TSS). The enrichment of this genomic region was significantly increased by antibodies specific for Tax and Sp1 compared with control IgG (Figure 4B). These findings underscore that Tax, in conjunction with Sp1, localizes near the –3000 bp region upstream of the *RGMA* TSS, synergistically boosting *RGMA* transcriptional activity and thereby significantly increasing *RGMA* mRNA expression.

### Reversal of neuronal cell damage induced by HTLV-1–mediated RGMa through inhibitory antibodies.

**Figure 5:**
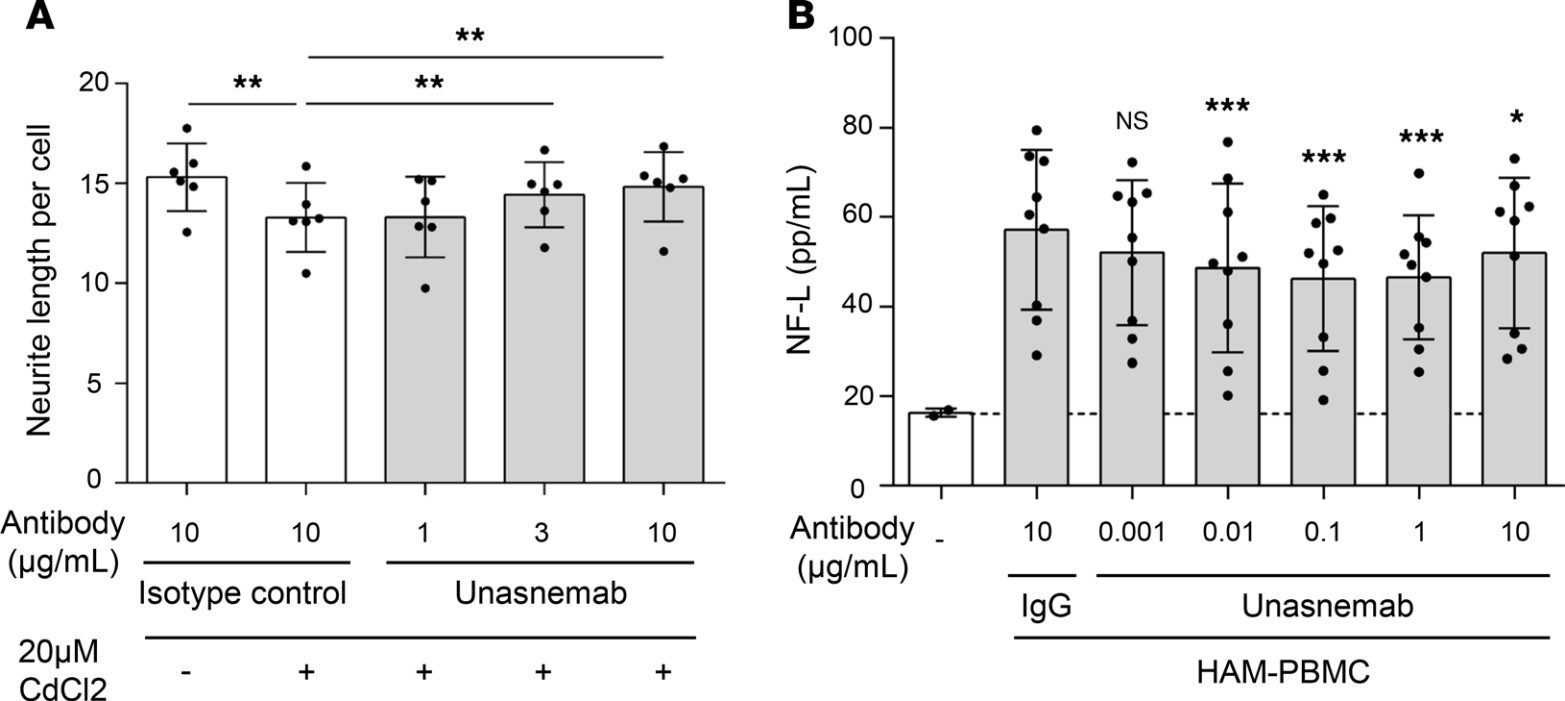
Neuronal cell damage mediated by RGMa. (**A**) Neurite length of cocultured NB-1 cells when exposed to Tax-inducing JPX9 cells treated with unasnemab in a dose-dependent manner for 48 hours. Palivizumab was used as a negative control. Data are shown as mean ± SEM (*n* = 6). Experiments were performed in triplicate. (**B**) The concentration of NF-L (pg/mL) in the supernatant when NB-1 cells were cocultured with HAM-PBMCs (*n* = 9) and with unasnemab in a dose-dependent manner for 72 hours. Normal human IgG (IgG) was used as a control. Data are shown as mean ± SD. *P < 0.05; **P < 0.01; ***P < 0.001 by 2-way ANOVA followed by Fisher’s least significant difference (LSD) post hoc test (**A**) or 1-way ANOVA with Dunnett’s multiple-comparison test (**B**). NF-L, neurofilament light chain.

To elucidate the contribution of RGMa to neuronal cell damage in the context of HTLV-1 infection, we assessed the neurotoxic effects of Tax expression induction in JPX9 cells on cocultured NB-1 human neuronal cells. We observed a significant reduction in the average neurite length of NB-1 cells cocultured with Tax-expressing JPX9 cells (*P* < 0.01, Figure 5A). This reduction in neurite length was alleviated in a dose-dependent manner by the treatment with unasnemab, a neutralizing antibody against RGMa (*P* < 0.01). Additionally, the evaluation of NF-L concentration in the culture medium of NB-1 cells cocultured with PBMCs from patients with HAM revealed an increase in NF-L levels, indicative of neuronal damage. This increase was significantly reversed by unasnemab treatment in a dose-dependent fashion, beginning at a concentration of 0.001 μg/mL (*P* < 0.05, Figure 5B), with human IgG serving as a negative control. These results provide strong evidence that HTLV-1–infected cells in HAM may induce neuronal damage through RGMa expression, highlighting the potential of RGMa inhibition as a therapeutic strategy for mitigating neuronal damage in HTLV-1–associated neurological disorders.

## Discussion

Our study presents compelling evidence that HTLV-1–infected cells in HAM can induce neuronal cell damage through the expression of RGMa. The observed reduction in neurite length of NB-1 cells cocultured with Tax-inducing JPX9 cells, coupled with the elevated concentration of NF-L in the culture medium, underscores the neurotoxic impact of HTLV-1–induced RGMa expression. Importantly, the reversal of these damaging effects by the anti-RGMa neutralizing antibody unasnemab highlights the critical role of RGMa in neuronal damage, suggesting its potential as a therapeutic target for ameliorating neuronal damage in HAM.

Historically, the mechanisms of neuronal damage in HAM have been hypothesized to involve direct neuronal infection, a molecular mimicry–based autoimmune response, and bystander damage mediated by inflammatory cytokines. However, none of these hypotheses had been experimentally supported (11). Utilizing NF-L as a biomarker for neuronal cell damage, our study experimentally demonstrated that HAM-PBMCs exert a direct neurotoxic effect. Notably, HTLV-1–infected cells in patients with HAM exhibited elevated expression of RGMa, a molecule known for its inhibitory effects on axonal regeneration, thereby suggesting a previously unrecognized mechanism of neuronal damage in HAM.

RGMa, a glycosylphosphatidylinositol-anchored protein expressed on the surface of oligodendrocytes and microglia, binds to a receptor complex comprising neogenin and unc-5 netrin receptor B (Unc5B) on neurons (19, 20). Through this interaction, RGMa exerts potent axonal growth–inhibitory effects by modulating various signaling pathways, including the activation of RhoA and inactivation of Ras (14). Beyond its role in the nervous system, RGMa is implicated in immune regulation, promoting helper T cell activation (21) and is expressed on Th17 cells of patients with multiple sclerosis (22). The discovery that HTLV-1 Tax protein induces RGMa expression adds a potentially important dimension to our understanding of HAM pathogenesis, highlighting the importance of Tax beyond its role in immune response modulation.

RGMa expression, known for its involvement in axonal guidance and neural tube closure during embryonic development, is typically suppressed postnatally. However, it has recently been discovered that within injured neural tissues, RGMa expression is induced in glial cells and immune cells, playing a crucial role in inhibiting neuronal regeneration and promoting neuronal cell death. However, the regulatory mechanisms of *RGMA* expression remain largely unexplored. Our study identified that in lymphocytes from healthy individuals, *RGMA* expression is repressed through H3K27 trimethylation upstream of the *RGMA* gene, while in patients with HAM, this methylation is removed, facilitating *RGMA* expression. HTLV-1 Tax is known to form a complex with the H3K27 acetyltransferase CBP/p300, amplifying the transcriptional activity of target genes (23). Thus, the interplay between oncogenic virus proteins and host histone modification machinery, as seen in HTLV-1, HPV, and EBV, suggests a viral strategy to manipulate host gene expression for viral survival (24). Given that patients with HAM have enhanced HTLV-1 *Tax* expression compared with ACs and patients with ATL (13, 25), and that HTLV-1 Tax, in cooperation with Sp1, boosts expression of *RGMA*, it is conceivable that HTLV-1 Tax localizes near the TSS of the *RGMA* gene at –2916 bp through Sp1, inducing epigenetic priming. We have previously demonstrated that infected cells in patients with HAM form a characteristic gene expression profile (26), and the results of this study suggest that epigenetic priming by HTLV-1 Tax may be one of the causes.

The dose-dependent mitigation of neurotoxic effects by unasnemab opens potential avenues for exploring the therapeutic use of RGMa inhibitors in HAM. Considering the high expression of RGMa in HTLV-1–infected cells and its correlation with neuronal damage, targeted therapies against RGMa could offer substantial clinical benefits to patients with HAM. This approach could be particularly beneficial in the early stages of the disease, where preventing the progression of neuronal damage is crucial. Furthermore, our findings raise important questions about the broader implications of RGMa in immune-mediated neurological disorders. The specific induction of RGMa by HTLV-1 Tax protein suggests a unique mechanism of neuronal damage in HAM. This could pave the way for future research to dissect the roles of viral proteins in neurodegenerative processes, potentially offering insights into other similar conditions.

However, the limitations of our in vitro study necessitate further research, including clinical trials, to validate our findings and assess the efficacy of RGMa-targeted therapies in vivo. We initiated a phase I clinical trial of unasnemab (MT-3921) for HAM patients in 2022 (ClinicalTrials.gov NCT05240612), to primarily evaluate safety and pharmacokinetics of MT-3921 and assess whether RGMa inhibition leads to a reduction in neuronal damage and symptom amelioration.

In conclusion, our study not only highlights the detrimental role of RGMa in HTLV-1–mediated neuronal damage but also opens potential therapeutic possibilities for HAM. Further exploration of RGMa’s role in HTLV-1 pathogenesis and its therapeutic targeting could lead to substantial advancements in the treatment of HTLV-1–associated neurological diseases.

## Methods

### Sex as a biological variable.

Both male (*n* = 6) and female (*n* = 15) donors were included in the study. The sex distribution of the enrolled participants is consistent with the known epidemiological trend of HAM, which affects females approximately 2 to 3 times more frequently than males. Owing to the limited sample size, sex-stratified analyses were not performed.

### Study participants and sample preparation.

The study included patients with HAM/TSP (*n* = 21, 6 male and 15 female; mean age, 65 years) and non–HTLV-1–infected (*n* = 10, 4 male and 6 female; mean age, 42 years). HTLV-1 seropositivity was determined using a particle agglutination assay (Serodia-HTLV-1, FUJIREBIO Inc.) and confirmed via Western blotting (SRL Inc.). HAM was diagnosed according to the World Health Organization guidelines (27). Diagnosis of ATL was based on the criteria established by Shimoyama (28). PBMCs were isolated using density gradient centrifugation (Pancoll, PAN-Biotech GmbH) and viably cryopreserved in liquid nitrogen with Cell Banker 1 (Mitsubishi Chemical Medience Corporation). CD4^+^ T cells were isolated from PBMCs using negative selection with magnetic beads (MACS CD4^+^ T cell isolation kit, Miltenyi Biotec).

### Cell culture conditions.

PBMCs from both HDs and patients with HAM/TSP were seeded at 1 × 10^5^ cells/well in 96-well round-bottom plates in RPMI 1640 medium (FUJIFILM Wako Pure Chemical Corporation), supplemented with 10% heat-inactivated FBS (GIBCO, Thermo Fisher Scientific), and 1% penicillin/streptomycin antibiotic solution (P/S) (FUJIFILM Wako Pure Chemical Corporation). NB-1 cells (RCB1953, RIKEN Cell Bank) were cultured in a medium consisting of 45% RPMI 1640 and 45% MEM, supplemented with 10% heat-inactivated FBS and 1% P/S. The cells were seeded in collagen-coated 24-well plates at a density of 4.4 × 10^4^ cells/well and cultured for 24 hours. Subsequently, PBMCs were added, and the cells were further cultured for 72 hours in the presence of mogamulizumab (KyowaKrin Co., Ltd.), or a humanized monoclonal antibody that binds to RGMa (unasnemab/MT-3921) (Mitsubishi Tanabe Pharma Corporation), or Human IgG1 kappa Isotype Control (Medical and Biological Laboratories Co., Ltd.). JPX9 is a subline of the Jurkat cell line carrying the *Tax* gene under the control of the metallothionein promoter (provided by Dr. Masataka Nakamura, Institute of Science Tokyo, Tokyo, Japan) (29). JPX9 cells were cultured in RPMI 1640 medium supplemented with 10% FBS, 1% P/S, and CdCl_2_ at a final concentration of 20 μM (NACALAI TESQUE, INC.) to induce the expression of HTLV-1 *Tax* over periods of 24, 48, and 72 hours.

### Measurement of NF-L.

NF-L is a neuron-specific structural protein and is a widely recognized clinical biomarker for various neurodegenerative diseases due to its release from damaged axons (30). The concentration of NF-L in the culture medium of NB-1 cells was determined using a sensitive sandwich ELISA method (Neurofilament, Light Polypeptide High Sensitive ELISA Kit; Cloud-Clone Corp.). The lower limit of quantification of the assay was 0.61 pg/mL.

### ChIP-on-chip experiments for H3K27me3.

The following procedures were performed according to the Agilent ChIP-on-chip protocol (v11.0) with slight modifications. CD4^+^ T cells isolated from patients with HAM, HDs, and patients with ATL were used in this study. CD4^+^ T cells (1 × 10^7^) were initially cross-linked in fixing solution. After washing, the cell pellets were resuspended in lysis buffers, and the nuclei were then incubated with micrococcal nuclease at 37°C followed by sonication for 10 minutes. The sheared chromatin was subsequently incubated with either Dynabeads anti–rabbit IgG or Dynabeads Protein G (Thermo Fisher Scientific) coated with 15 μg of a ChIP-grade anti-H3K27me3 antibody (07-449, Merck Millipore) or control IgG (2729S, Cell Signaling Technology) overnight at 4°C. The antibody-protein complexes were collected, washed, and eluted by SDS-containing buffer. The samples were incubated overnight at 65°C to reverse cross-links and were then treated with RNase A and Proteinase K. The purified DNA was treated with T4 DNA polymerase and then ligated with linker. A 2-step linker-mediated PCR was performed and each amplified DNA was used to fluorescent labeling. The labeled ChIP sample and the input chromatin were hybridized to SurePrint G3 Human Promoter Microarrays (Agilent Technologies). After hybridization and scanning, initial data mining was performed on GeneSpring 12.5 (Agilent Technologies). We further selected data entities based on their signal intensity values to remove very low signal values, and then performed normalization (75th centering based on data distribution) within a batch, allowing comparison among arrays (recommended by Agilent technologies). We further normalized by removing the any variations based on standard deviation (SD).

### GEO gene expression data.

Gene expression profiling of patients with HAM and ATL, HTLV-1 carriers, and normal CD4^+^ T cells has been performed previously. The coordinates have been deposited in the NCBI Gene Expression Omnibus database (GEO GSE55851 and GSE233437) (26, 31). The GSE233437 dataset includes transcriptomic data from CD4^+^ T cells derived from non–HTLV-1–infected HDs (*n* = 4) and patients with HAM (*n* = 4). The GSE55851 dataset includes data from HTLV-1–infected cells derived from ACs (*n* = 2), smoldering ATL (*n* = 2), chronic ATL (*n* = 1), and acute ATL (*n* = 3). H3K27me3 data for ATL and normal T cells have been deposited (GSE71450, primary ATL cells with high proviral load [>100%] and resting CD4^+^ T cells isolated from HDs) (32). These datasets were reanalyzed using GeneSpring 12.5 software (Agilent Technologies). For data analysis, entities were selected based on their signal intensity values (removing very low signals). The data were normalized by 50th-percentile centering of intensity distributions of all samples within a batch and by removing any variations based on SD.

### Preparation of Tax-expressing cells.

HTLV-1 *Tax* cDNA was cloned into the CSII-EF1a-IRES-Venus lentiviral vector (32). Jurkat cells were infected with the lentiviral vector and cultured in RPMI 1640 with 10% heat-inactivated FBS. Venus-positive cells were detected by FACSCalibur (BD Biosciences). RNA was subsequently extracted, and cDNA was prepared according to a previously described method (33).

### Plasmids.

The *RGMA*-Luc reporter gene plasmid was constructed by amplifying a region spanning –3500 bp upstream to +100 bp downstream of the TSS of the human *RGMA* gene (NM_001166283) from human PBMC genomic DNA using PCR. This PCR product was then digested with KpnI and XhoI restriction enzymes and cloned into the pPicaGene-Basic Vector II (Toyo-ink.). Previously reported Tax, Tax C29A, and Sp1 expression plasmids were utilized (13, 18, 32). The primer sequences used for PCR were as follows: 5′-CGCGGTACCGGGCAGAAACTGACAGACCTG-3′; 5′-CGCCTCGAGCAGAGAGATGGCTATGCCGGTC-3′.

### Luciferase assay.

For the luciferase assay, HEK293 cells (RCB1637, RIKEN Cell Bank) were seeded at a density of 5 × 10^4^ cells per well in 24-well collagen-coated plates using MEM supplemented with 10% heat-inactivated FBS and 1% P/S. After 24 hours, cells were transiently transfected with each plasmid using Lipofectamine 2000, following the manufacturer’s protocol (Thermo Fisher Scientific). To normalize transfection efficiency, 50 ng of the pRSV-β-gal plasmid was included in each experiment. The total DNA quantity in all samples was standardized with pcDNA3. Forty-eight hours after transfection, cells were lysed with Passive Lysis Buffer (Promega), and luciferase activity was measured using the Promega Luciferase Assay System and GentroXS3 LB960-Dual (Berthold Technologies), with values normalized to β-gal activity for internal control.

### Real-time qPCR analyses.

Total RNA isolation was performed using Sepasol (NACALAI TESQUE, INC.). In the experiment analyzing *Tax*, *HBZ*, and *RGMA* gene expression levels in cultured HAM-PBMCs, mRNA was purified using the Magnetic mRNA isolation kit (New England BioLabs). The isolated RNA underwent reverse transcriptase (RT) reactions using the ReverTra Ace qPCR RT Master Mix with gDNA Remover (TOYOBO) following the manufacturer’s protocol. Real-time qPCR was conducted using ABI Prism 7500 SDS (Applied Biosystems), FastStart Universal Probe Master (ROX) (Roche Diagnostics), and human FAM-labeled TaqMan gene expression primers for the following genes: *RGMA* (Hs00297192_m1, Applied Biosystems) and Eukaryotic *18S rRNA* (4319413E, Applied Biosystems). Additionally, the expression levels of HTLV-1 *Tax*, and *HBZ* mRNA were quantified via qPCR using PowerUp SYBR Green Master Mix (Applied Biosystems). Relative quantification of mRNA expression levels was performed using the comparative threshold cycle method using Eukaryotic *18S rRNA*, *GAPDH*, and ribosomal protein L19 (*RPL19*) as endogenous controls. Target gene expression was normalized to the expression of Eukaryotic *18S rRNA*, *GAPDH*, and *RPL19* for each sample. The following formula was used to calculate the relative expression levels: target gene expression = 2^–(Ct^ ^[target]^^–^^Ct^ ^[endogenous^ ^control])^. The primer sequences using SYBR Green are as follows: *Tax*-F: 5′-CCGGCGCTGCTCTCATCCCGGT-3′, *Tax*-R: 5′-GGCCGAACATAGTCCCCCAGAG-3′; *HBZ*-F: 5′-TAAACTTACCTAGACGGCGG-3′, *HBZ-*R: 5′-CTGCCGATCACGATGCGTTT-3′; *RGMA*-F: 5′-CAACACGCCTGTGCTGCCCG-3′, *RGMA*-R: 5′-CCACCGTTCTTAGAGCCATCCA-3′; *GAPDH*-F: 5′-AGCCACATCGCTCAGACA-3′, *GAPDH*-R: 5′-GCCCAATACGACCAAATCC-3′; *RPL19*-F: 5′-ACCAAGGAAGCACGCAAGC-3′, *RPL19*-R: 5′-CAGACAAAGTGGGAGGTTTTATTTC-3′.

### ChIP-qPCR.

ChIP assays were performed using the SimpleChIP Enzymatic Chromatin IP Kit (Cell Signaling Technology) according to the manufacturer’s instructions. Briefly, JPX9 cells (4 × 10^6^) incubated in 20 μM CdCl_2_ for 48 hours were fixed with 1% formaldehyde for 10 minutes at room temperature, quenched in glycine solution, and then washed in cold PBS. Nuclei extracted with lysis buffer were digested by micrococcal nuclease for 20 minutes at 37°C and sonicated for 30 seconds on and 30 seconds off for 10 minutes using a Bioruptor UCD-250 (Diagenode) to break the nuclear membrane. Immunoprecipitation of extracted chromatin was performed using 2 μg of anti-Tax (Lt-4; gifted by Yuetsu Tanaka, University of the Ryukyus, Okinawa, Japan) (34) and 2.6 μg of Sp1 (D4C3, Cell Signaling Technology) antibodies. Normal mouse IgG and rabbit IgG (5415S and 3900S, both from Cell Signaling Technology) were used as negative controls, respectively. Purified ChIP samples were subjected to qRT-PCR using QuantStudio Real-Time PCR (Applied Biosystems) and THUNDERBIRD SYBR qPCR Mix (TOYOBO). The corresponding input DNA values were used to normalize the qPCR results. The following formula was used to calculate the enrichment of the target regions: percentage of input = 2^(Ct^ ^[input]^^–^^Ct^ ^[ChIP^ ^sample])^ × 100. The primer sequences are as follows: 3000 bp upstream from the TSS at *RGMA* locus-F: 5′-TCCTACACTGGGAAAGCTTCTT-3′; 3000 bp upstream from the TSS at *RGMA* locus-R: 5′-CTTAAAGTCAAGGCGAGACAGA-3′.

### Flow cytometric analysis.

Normal goat IgG (AB-108-C, R&D Systems) and RGMa (AF2459, R&D Systems) antibodies were biotinylated using the Biotin Labeling Kit-NH2 (Dojindo Laboratories). Biotinylated Normal goat IgG or biotinylated anti-RGMa antibody was incubated with PBMCs along with cell surface marker antibodies anti-CD3 BV510 (UCHT1, Biolegend), anti-CD4 APC (OKT4, eBioscience), and anti-CCR4 PE (1G1, BD Biosciences), and after washing, biotinylated normal goat IgG and anti-RGMa antibodies were stained with BV421 streptavidin (405226, Biolegend). JPX9 cells were incubated with biotinylated normal goat IgG or biotinylated anti-RGMa antibody, and after washing, stained with BV421 streptavidin. Subsequently, cells were permeabilized and fixed with Fixation/Permeabilization Concentrate and Diluent (eBioscience) and intracellularly stained with anti-Tax FITC (Lt-4) (34). Zombie NIR Fixable Viability Kit (BioLegend) was used to stain the dead cells.

### Measurement of neurite length.

JPX9 cells, treated with 20 μM CdCl_2_ for 48 hours and NB-1 cells, seeded 24 hours prior, were cocultured with unasnemab at concentrations of 1, 3, and 10 μg/mL. Palivizumab (AbbVie) was used as an isotype control antibody. After coculturing Tax-induced JPX9 and NB-1 cells for 48 hours, the cells were fixed with 4% paraformaldehyde (Alfa Aesar) and 1% glutaraldehyde (FUJIFILM Wako Pure Chemical Corporation), treated with 0.2 M glycine solution to inactivate the unreacted aldehyde groups, and then permeabilized with 0.2% Triton X-100 (Sigma-Aldrich, Merck). After blocking with normal goat serum (Thermo Fisher Scientific) and FcR blocking reagent (Miltenyi Biotec) in a 4:1 ratio, anti-MAP2 antibody (ab5392; Abcam) and fluorescence-conjugated anti–chicken IgY antibody (A11039, Thermo Fisher Scientific) were used as a primary and secondary antibody, respectively. Nuclei were stained with 2 μg/mL Hoechst (Dojindo Laboratories). Fluorescence images were acquired using an imaging cytometer (IN Cell Analyzer 2500HS, Molecular Devices), and the neurite length per neuronal cell body was automatically calculated using the Developer Toolbox (v1.9.2, Molecular Devices). Imaging was performed in the same field of view across all wells, with a total of 9 fields analyzed.

### Statistics.

To compare 2 groups, an unpaired 2-tailed Student’s *t* test was employed. For multiple comparisons, a 1-way ANOVA was used, followed by Dunnett’s test. Statistical analyses and the generation of graphs were carried out using GraphPad Prism 7 (GraphPad Software). Statistical significance was set at a *P* value of less than 0.05.

### Study approval.

This study was approved by the Bioethics Committee of the St. Marianna University School of Medicine (approval ID no. 1646). Written informed consent was obtained from all participants before the study.

### Data availability.

The gene expression data and the ChIP-on-chip data have been deposited in the NCBI GEO under the accession numbers GSE55851, GSE233437 (gene expression microarray), and GSE71450 (ChIP-on-chip data). Values for all data points are reported in the Supporting Data Values file.

## Author contributions

NA, MY, MN, NA, KK, TS, and YY designed the research studies. NA, MY, MN, NA, KK, and II conducted experiments. NA, MY, MN, NA, KK, SA, and II acquired data. NA, MY, MN, NA, KK, SA, II, KU, and YY analyzed data. NA, KK, NY, EH, TW, TS, KU, and YY provided resources. NA, MY, MN, NA, KK, and YY wrote the manuscript. The order of the authors was determined based on the relative degree of effort contributed to the study, including experimental work, data acquisition, analysis, and manuscript preparation. All authors read and approved the final manuscript.

## Supplementary Material

Supplemental data

Unedited blot and gel images

Supporting data values

## Version 1 04/30/2025

In-Press Preview

## Version 2 06/09/2025

Electronic publication
