# Supplementary material for: Virus-induced RGMa expression drives neurodegeneration in HTLV-1–associated myelopathy
Source: JCI Insight. 2025 Apr 24;10(11):e184530. doi: 10.1172/jci.insight.184530 (PMC12220939; doi:10.1172/jci.insight.184530)
Supplement: Supplemental data [file jciinsight-10-184530-s061.pdf]

A

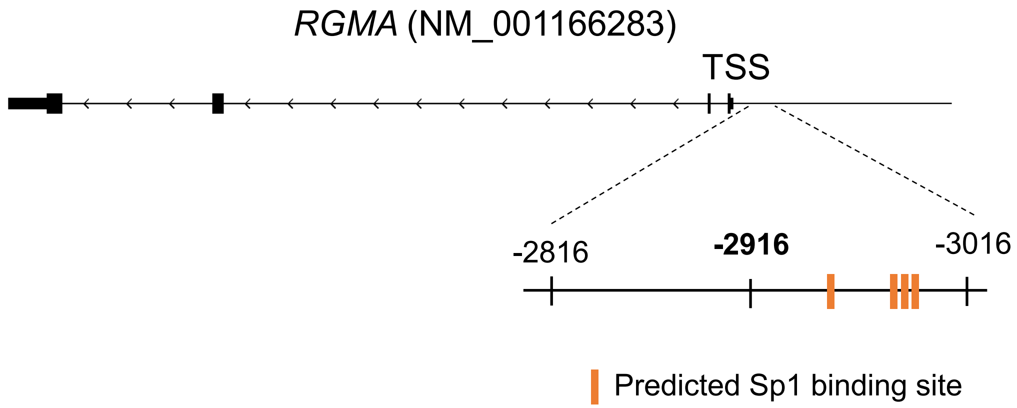

B

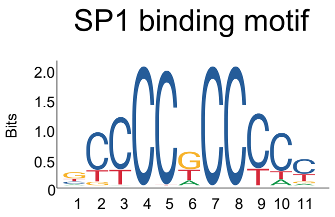

Predicted sequence	Start	End	Strand	Relative score (%)
TCCCCTCCCTG	-3007	-2997	+	91.3
AGGGAGGGG	-3006	-2998	-	85.8
GCTCCAGCCCA	-2986	-2976	-	81.2
GGGGTGCAG	-2999	-2991	-	80.9

Supplementary Figure

Predicted binding of Sp1 in the transcriptional regulatory region of the *RGMA* gene. (A) Schematic illustration of the predicted Sp1 binding site in near the transcription start site (TSS) of the *RGMA* gene. (B) Motif plot for Sp1 (Left). JASPAR analysis results for Sp1 binding sequences located near the TSS of *RGMA* gene at -2916bp (Right). TSS; transcription start site
