# Supplementary figures and images for: Virus-induced RGMa expression drives neurodegeneration in HTLV-1–associated myelopathy
Source: JCI Insight. 2025 Apr 24;10(11):e184530. doi: 10.1172/jci.insight.184530 (PMC12220939; doi:10.1172/jci.insight.184530)

Full unedited gels for Figure 3B

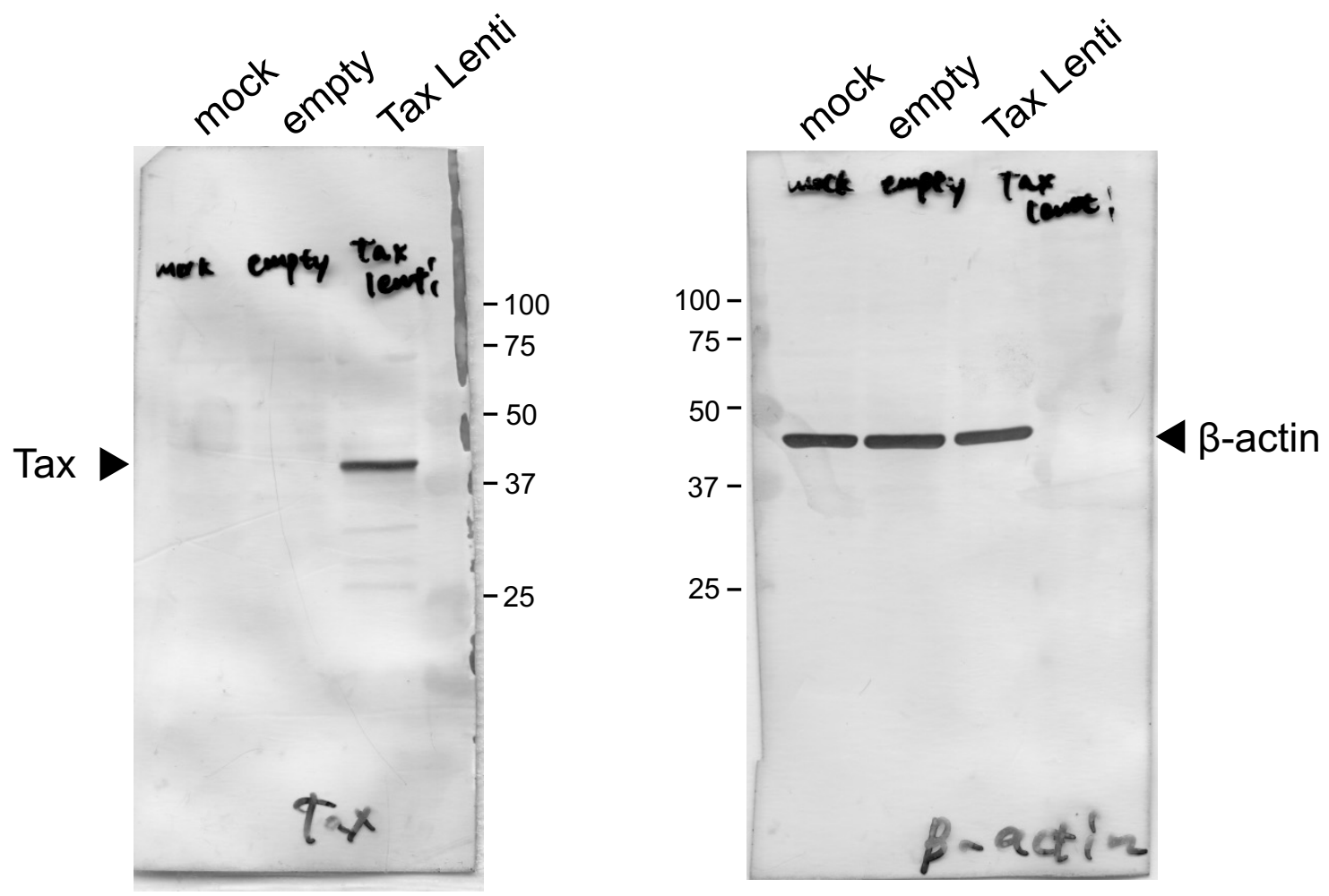

Supplement: Unedited blot and gel images [file jciinsight-10-184530-s062.pdf]
